# Mental capacity assessment in the multi-professional real world: a qualitative study of six areas of uncertainty

**DOI:** 10.12688/wellcomeopenres.20952.1

**Published:** 2024-04-24

**Authors:** Andrew McWilliams, Kevin Ariyo, Anthony S. David, Gareth S. Owen

**Affiliations:** 1 Mental Health, Ethics and Law Research Group, Department of Psychological Medicine, King's College London, London, SE5 8AF, UK; 2 Metacognition Group, Wellcome Centre for Human Neuroimaging, University College London, 12 Queen Square, London, WC1N 3BG, UK; 3 Child and Adolescent Mental Health Services, Great Ormond Street Hospital for Children NHS Foundation Trust, Great Ormond Street, London, WC1N 3JH, UK; 4 UCL Institute of Mental Health, Department of Psychiatry, University College London, London, W1T 7NF, UK

**Keywords:** Mental capacity, capacity assessment, court of protection, framework analysis, undue influence, neuropsychological assessment, remote assessment

## Abstract

**Background:**

The Mental Capacity Act 2005 of England and Wales is a ground-breaking piece of legislation with reach into healthcare, social care and legal settings. Professionals have needed to develop skills to assess mental capacity and handle malign influence, but it is unclear how assessments are implemented in real world settings. Our previously reported survey found professionals juggling competing resources in complex systems, often struggling to stay up to date with law.

The current follow-up study uses one-to-one interviews of professionals to characterise in detail six areas of uncertainty faced when assessing mental capacity, whilst suggesting ways to make improvements.

**Methods:**

Forty-four healthcare, social care and legal professionals were interviewed, using a semi-structured topic guide. Transcripts were analysed using framework analysis: a qualitative technique built to investigate healthcare policy.

**Results:**

Our topic guide generated 21 themes. In relation to the six areas of uncertainty: 1) Many participants stressed the importance of capturing a holistic view, adding that their own profession was best-placed for this - although a medical diagnosis was often needed. 2) The presumption of capacity was a laudable aim, though not always easy to operationalise and occasionally being open to abuse. 3) There was cautious interest in psychometric testing, providing a cognitive context for decisions. 4) Undue influence was infrequent, but remained under-emphasised in training. 5) Multi-professional assessments were common, despite doubts about fitting these within local resources and the law. 6) Remote assessment was generally acceptable, if inadequate for identifying coercion.

**Conclusions:**

Practical constraints and competing demands were reported by professionals working within real world systems. Assessment processes must be versatile, equally applicable in routine and emergency settings, across diverse decisional types, for both generalist and specialist assessors, and able to handle coercion. Recognising these challenges will guide development of best practices in assessment and associated policy.

## Plain language summary

The Mental Capacity Act 2005 of England and Wales is an important piece of law for professionals working in health and social care or as lawyers. It explains how to assess whether a person is able to take a particular decision for themselves – and therefore has “mental capacity” in the eyes of society. Professionals have needed to develop skills to assess mental capacity, and to recognise situations where family or friends are trying to influence decisions for their own interests.

We previously reported a large scale survey of professionals who assess mental capacity. The current study uses one-to-one interviews, exploring in detail six areas of uncertainty around capacity assessment which were described in that survey.

We interviewed 44 health and social care professionals and lawyers, then analysed their responses. We found 21 themes relating to the six areas of uncertainty: 1) The importance of capturing a holistic view of the person’s life; 2) Challenges for assessors when trying to presume initially that a person does have mental capacity, as the law asks assessors to do; 3) Detailed testing by psychologists could be useful; 4) Other people were rarely thought to try to influence decisions, but awareness of this possibility should be emphasised in training; 5) Multi-professional assessments were common, despite doubts about whether these were possible in all settings or fit with the law; 6) Remote assessment was generally acceptable, but was poor at identifying if people were being influenced.

Practical constraints and competing demands were reported by professionals working within real world systems. Our analysis suggests that mental capacity assessment processes need to be versatile, so they can work well in routine and emergency settings and for a range of types of decisions. Recognising these challenges will guide development of best practices in assessment and associated policy.

## Introduction

The Mental Capacity Act of England and Wales 2005 (MCA 2005) is a ground-breaking piece of statute legislation, attempting to balance society’s wish to promote personal autonomy against that of protecting best interests. To be declared as lacking mental capacity, a person must (at s.2 MCA 2005) have ‘an impairment of brain or mind’, leading to an impairment in decisional functioning (at s.3). Decisional functioning is itself determined by assessing 4 abilities: understanding of relevant information, retaining that information, using or weighing the information to reach a choice, and communicating that choice. Additionally, the act begins by laying out some overarching principles (s.1 MCA 2005). These include that people must be presumed to have capacity unless it can be shown otherwise, as well as that “all practicable steps” must be taken to support the person at the time of the assessment to help them demonstrate that they have capacity. A person cannot be found to lack capacity solely because they have made an unwise decision. Anyone found to lack capacity may then have decisions taken on their behalf, following principles of “best interests”. The MCA 2005 created a specialised court - the re-established Court of Protection (CoP) - to arbitrate on contested capacity and best interests disputes, as well as to rule on points of law related to the MCA 2005 itself ( Ruck Keene *et al.*, 2019; Weston, 2020).

Since implementation of the act in 2007, a wealth of training has been delivered to the various professionals who assess mental capacity for health, welfare and financial decisions. However, human rights scholars have expressed concern about the variability in approaches used to assess capacity ( Szmukler, 2017). Some research has found assessment practices to have the good measurement properties which we would look for in any assessment procedure ( Okai *et al.*, 2007). For example, when assessing capacity to consent to treatment in 55 recently admitted psychiatric in-patients, there was high interrater reliability, both when 2 different assessors carried out separate interviews with the same patient or when clinicians watched a filmed interview and formed independent views ( Cairns *et al.*, 2005). However, other empirical work has found more modest reliability, such as with experienced psychiatrists assessing capacity to consent to take part in research in 188 people with Alzheimer’s ( Kim *et al.*, 2011). Yet the development of a standardised – or even psychometric – mental capacity assessment tool will only help assessors if it is sufficiently sensitive to contextual factors in real lives and can offer a determinative outcome ( Breden & Vollmann, 2004; Kapp & Mossman, 1996). So although structured assessment tools exist for use in research studies ( Candia & Barba, 2011; Ghesquiere *et al.*, 2019; Lamont *et al.*, 2013; Okai *et al.*, 2007), they have yet to prove useful in the clinic or appear in the CoP ( McWilliams *et al.*, 2020), although it has been argued they would be of interest to the courts ( Donnelly, 2009).

The MCA 2005 also describes the importance that decisions are taken freely and voluntarily, and not under duress, coercion or undue influence from other parties. This notion of undue influence predates the MCA 2005, but it is still taking shape among scholars of law and medical ethics ( Craigie, 2015; Craigie, 2021). Several years after the MCA 2005 was implemented, the courts confirmed that they could not safeguard all adults who were subject to undue influence and those who have capacity can only be protected under a different legal framework: the high court’s inherent jurisdiction (see *DL v A Local Authority & Ors* [2012] EWCA Civ 253). Since then, professionals have brought several cases before the courts, while concerned about both lack of capacity and undue influence. In the most complex capacity cases, it has proven difficult even for the courts to distinguish between benign and malign influence.

Human rights organisations have also strongly advocated that people are supported by those who matter to them when making decisions, rather than to have professionals or others make decisions on their behalf ( Devi *et al.*, 2011; Gooding, 2013). However, the United Nations Convention on the Rights of Persons with Disabilities (UNCRPD) also made clear that decisions must be ‘free of conflict of interest and undue influence’ ( Szmukler *et al.*, 2014). This raises novel questions about how to best facilitate the involvement of families and carers without negative repercussions. Many scholars have argued that, in real life scenarios, supporters may be projecting their own interests onto the person they are trying to support, in a way that might reduce autonomy, even if done inadvertently or with the best intentions ( Kong, 2017; Scholten & Gather, 2018). Different professions vary in the extent to which law, including human rights law, features in their training, and there remains a question as to how undue influence, and relationships more generally, are understood and navigated during capacity assessments. Few studies have explored how healthcare professionals consider undue influence.

Since implementation of the MCA 2005, some research reports on how well professionals know and understand mental capacity law ( Willner *et al.*, 2011), but few studies characterise how that knowledge is put into practice or ask professionals where improvements could be made. Studies about assessment practices have tended to gather small, uniprofessional samples or be restricted to those in particular settings. For example, there are small studies of volunteers ( Manthorpe *et al.*, 2012), community dementia nurses ( Samsi *et al.*, 2012) and clinical psychologists ( Walji *et al.*, 2014), and of professionals who work with older adults ( Hinsliff-Smith *et al.*, 2017), many of whom reported feeling unable to put the law into practice. The importance of building inter-agency links, sharing resources and promoting awareness of mental capacity to the general public has been suggested ( Manthorpe *et al.*, 2014). Psychiatrists have found time-constraints a significant concern for assessors ( Shah *et al.*, 2010), a finding echoed in our own work ( Ariyo *et al.*, 2021), as well as discussion about how closely mental capacity law serves its stated purposes ( Sawhney *et al.*, 2009).

The current disparate literature makes it is hard to gain a multi-disciplinary overview of assessment practices, with some studies carried out rather soon after implementation of the act in 2007, when training and practice would not have bedded in. The available literature also does not deal with some important specific issues. For example, during the height of the COVID-19 pandemic, remote assessments of capacity were confirmed as lawful by Hayden J ( *BP v Surrey County Council & Anor* [2020] EWCOP 17), and in April 2020, some use of remote assessment in mental health was also supported within the National Health Service ( NHS London Clinical Networks, 2020). Little research looks at remote delivery of capacity assessment, despite obvious concerns about how to support the person online with s.1 MCA 2005 during assessment and how to handle third parties who could be are present off-camera. Some initial work has elucidated concerns by those assessing capacity remotely in care homes, including around establishing the relevant information needed for a particular decision ( Kuylen *et al.*, 2022).

We previously published an exploratory survey of 611 professionals, who told us about a variety of issues: competition for resources, a desire for more training, hints about the usefulness of structured tools, and some concern about assessing capacity when they suspect undue influence ( Ariyo *et al.*, 2021). That study involved predominantly social workers, and our findings were limited by the survey format short responses. We therefore planned a follow-up interview study across a more balanced range of professionals, exploring six areas we knew attracted uncertainty to suggest ways in which processes and policy might be improved.

## Methods

### Participants

Participants were recruited between April and September 2021. The breakdown of professional groups was determined using quota sampling, aiming for approximately 40 participants equally spread across five main professional groups who assess capacity in their practice: clinical psychologists, psychiatrists, other doctors, social workers and lawyers. Initial recruitment took place by contacting responders to our previous exploratory survey who had indicated that they might be interested to take part in further research ( Ariyo *et al.*, 2021). In the lawyer and non-psychiatrist doctor groups, recruitment was then supplemented by contacting professionals suggested by other interview participants (the “snowballing” method ( Streeton *et al.*, 2004)) in order to allow us to reach our sample targets. A small number of types of other professionals were also included, being survey participants who had indicated an interest take part in further research, although their recruitment was not prioritised as the five principal groups already covered a wide breadth of perspectives. Written informed consent was received for participation, supplied electronically and in line with our ethical approval.

### Procedure

The data collection and analysis were led by 2 primary researchers: AMcW (a specialist registrar in psychiatry, mental capacity researcher and ex-mental health nurse with an undergraduate psychology degree) and (KA: a research psychologist with a background in cognitive psychology and mental capacity research). The study team also included regular reflexive review by 2 senior researchers: AD and GO (general adult psychiatrists with expertise in liaison and neuropsychiatry, mental capacity and insight).

Interview participants were randomly allocated to one of the two primary researchers, with members of each professional group divided between the two researchers evenly. The interview topic guide contained 7 questions generated from themes arising from our previous study ( Ariyo *et al.*, 2021), the wider literature, the MCA 2005 itself and our anecdotal clinical experience. Interviews were one-to-one, lasting between 20 and 60 minutes, and following a semi-structured format where the researcher could ask spontaneous follow-up questions for clarification or elaboration. We aimed to explore uncertainties in the following six areas of assessment: 1) profession-specific competencies, 2) presumption of capacity, 3) use of psychological tests, 4) undue influence, 5) multi-disciplinary team involvement, 6) remote assessment (see Table 1).

**Table 1.** Interview topic guide. The one-to-one interviews of professionals were carried out following this semi-structured topic guide. The researcher used it to lead a discussion of topics taken from our previous research, the wider literature and the law. This explored how professionals assess mental capacity, where they see the obstacles to best practice to be and how they thought these might be overcome.

Topic	Question/Prompt
*1. Profession-specific* *issues*	What are the particular strengths and disadvantages or problems of being a *[insert name of* *profession]* when assessing capacity, compared to those faced by members of other professions?
*2. Presumption of* *capacity*	What do you understand by the principle of presumption of capacity?
*3. Structured testing*	Standard psychological tests are often used to demonstrate that someone has an impairment of brain or mind, but could it be helpful to use psychological, psychometric or other structured testing to assess decisional functional abilities, and if so, how?
*4. Undue Influence (1)*	In your experience, how do you assess for the presence of undue influence, and do you feel that enough attention is paid to this?
*5. Undue Influence (2)*	If you suspect that someone is being unduly influenced, how might you try to support the person during a capacity assessment?
*6. Responsibility and* *sharing assessment*	Would it be possible or desirable for assessment of mental capacity to be made jointly by particular teams of professionals, with each member playing a role in the assessment, but given the fact that they may ultimately not agree on the outcome?
*7. Remote assessment*	And finally, I want to ask about remote assessment of mental capacity, such as by telephone or web camera, though I appreciate that you may or may not have used these methods yourself. Remote assessment of any potentially vulnerable person will pose a number of challenges for delivery of an optimal assessment, both for the assessor and the assessee. Are there issues which are unique to remote assessment of capacity in particular, over and above those faced in remote assessments of other kinds with vulnerable people?

### Analysis

Interviews were initially transcribed using an automated transcription service ( https://www.descript.com), in line with our ethical approval. AMcW and KA then corrected the transcripts by ear as necessary, ensuring the researchers were immersed in the data ( Braun & Clarke, 2006). The transcripts were then used to create seven datasets (one for each interview question), which were each analysed using framework analysis ( Ritchie & Spencer, 1993; Ritchie *et al.*, 2003). This qualitative analysis technique was developed specifically to answer research questions around policy and its implications. It also has a strong track record in both healthcare and psychological research ( Furber *et al.*, 2009; Pope *et al.*, 2000; Samsi *et al.*, 2012; Ward *et al.*, 2013).

The 5 steps of the framework approach (familiarisation with the data; identification of a thematic framework; indexing data; charting the responses; mapping and interpretation ( Ritchie & Spencer, 1993)) were applied in an iterative manner. The processing of new data involved returning to check for goodness of fit with the themes, which were updated as necessary. Our final set of themes, framed by our topic guide questions, are presented with verbatim quotes, which illustrate their conceptual meaning to the framework while also preserving their original context.

### Ethical approval

Ethical approval was achieved from the King’s College London’s College Research Ethics Committee on 11/02/19 (MRA18/19-10500).

## Results

### Participants

There were 44 participants: 8 social workers, 8 clinical psychologists, 8 psychiatrists, 7 non-psychiatrist doctors (4 general practitioners, 1 intensivist, 1 obstetrician/gynaecologist, 1 general physician), 7 lawyers and 6 other health professionals (3 nurses, 2 occupational therapists and 1 speech and language therapist). The sample covered a spectrum of degrees of interest in mental capacity, so although many were practice- or research-specialists in mental capacity, others had no such interest – beyond being curious enough to agree to participate in the study.

### Framework analysis results

The thematic structure of the 21 themes (3 for each question) is shown ( Table 2). A detailed description follows, incorporating participant quotations.

**Table 2.** Thematic structure. Framework analysis was used to analyse the interview data – a qualitative technique designed to explore questions about health and social care policy. The research team analysed the transcripts of the 44 professionals, deriving 21 themes about mental capacity and its assessment (3 for each of the 7 interview topics).

	Theme
***Question 1**. Profession-specific issues* What are the particular strengths and disadvantages or problems of being a *[insert name of profession]* when assessing capacity, compared to those faced by members of other professions?	
1a	Not all mental capacity training is the same
1b	It’s harder if you don’t know about diagnoses and medical jargon
1c	My profession gets to know the whole person
***Question 2**. Presumption of capacity* What do you understand by the principle of the presumption of capacity?	
2a	It reminds me that all decisions are different
2b	But I’m only assessing them as I think they might not have capacity!
2c	I do a low intensity assessment but then get an expert in
***Question 3**. Structured testing* Standard psychological tests are often used to demonstrate that someone has an impairment of brain or mind, but could it be helpful to use psychological, psychometric or other structured testing to assess decisional functional abilities, and if so, how?	
3a	They paint a useful picture of the cognitive context
3b	They can’t get at the actual legal criteria about decision-making
3c	I leave that to the psychologists
***Question 4**. Undue Influence (1: presence)* In your experience, how do you assess for the presence of undue influence, and do you feel that enough attention is paid to this?	
4a	It’s our prejudices which draw the line at what becomes undue
4b	Capacity assessment doesn’t involve assessment of undue influence
4c	I think about it most for financial decisions
***Question 5**. Undue Influence (2: support)* If you suspect that someone is being unduly influenced, how might you try to support the person during a capacity assessment?	
5a	I always see people alone
5b	I gather as much information as possible
5c	I haven’t thought about it much…where’s the guidance on it?
***Question 6**. Responsibility and sharing assessment* Would it be possible or desirable for assessment of mental capacity to be made jointly by particular teams of professionals, with each member playing a role in the assessment, but given the fact that they may ultimately not agree on the outcome?	
6a	Isn’t pooling knowledge, views and responsibility what we already do?
6b	You shouldn’t pretend you can diffuse responsibility for decision-making
6c	Nice idea, but it wouldn’t work when time is of the essence
***Question 7**. Remote assessment* And finally, I want to ask about remote assessment of mental capacity, such as by telephone or web camera, though I appreciate that you may or may not have used these methods yourself. Remote assessment of any potentially vulnerable person will pose a number of challenges for delivery of an optimal assessment, both for the assessor and the assessee. Are there issues which are unique to remote assessment of capacity in particular, over and above those faced in remote assessments of other kinds with vulnerable people?	
7a	It has its limits, but don’t assume all vulnerable people dislike technology
7b	I can meet them more than once now
7c	It’s tricky to pick up on environmental cues or coercion

***Question 1. Profession-specific issues***

**Theme 1a. Not all mental capacity training is the same**

Participants described several differences in training and skills between professional groups, which were seen as aligning with the core values and practices of that profession. For example:

*“So, I think social work training does equip you. You get used to dealing with those borderlines where things are fuzzy and grey and mixed and confusing and complex. So, I don’t always look for simple answers or simple yes or no’s. […] the principles of social work and the responsibilities of it and how we should practice, which is: taking each case individually to promote people’s human rights to respect and understand diversity and individual experience; that should set you up pretty well.”* Social worker

And,

*“I would say that probably other professions would say that capacity isn’t just like taking someone’s temperature. You can’t just say yes, they’ve got capacity to do this or that. I’m not saying that psychologists have gotten more sophisticated understanding, but we have got the potential to draw on quite a rich, theoretical background to understand how these categories – like capacity – how they evolve, and what sort of status they have.”* Clinical psychologist

**Theme 1b. It’s harder if you don’t know about diagnoses and medical jargon**

For some, capacity assessment was made harder by limits in professionals’ understanding of physical and mental healthcare language and medical diagnostic terminology. For example, when describing delivering mental capacity training to nurses, a social worker observed that:

*“…their deeper understanding of some of the medical issues was reflected in a better performance on writing reports that required people to comment on that.”* Social worker

There were even concerns, especially from lawyers, about whether all parts of the MCA 2005 test could be handled by a professional who does not have diagnostic skills.

*“The drawback was that, because we are legal professionals and not healthcare professionals, when it comes to the diagnostic test, I am wholly beholden to a psychiatrist or different other clinical professional who will tell me what the reality of having a diagnosis of, for example, schizophrenia would be, and what the implications of that are.”* Lawyer

**Theme 1c. My profession gets to know the whole person**

Participants from a range of professions said their own discipline was particularly well-placed to garner the desired holistic and person-centred view necessary for a high quality assessment.

*“I think GPs* [general practitioners] *have a huge advantage. I’ve lots of discussions with lots of different professionals, and social workers were always very dismissive of GPs capacity assessments. And I think that’s wrong, because I think GPs do know their patients well. And even if they don’t know them that well, they have their global health record.”* Doctor (non-psychiatrist)

Others discussed that this led to their profession being better placed than others for specific tasks, such as to consider a person’s likelihood of regaining capacity:

*“I think psychologists take quite a holistic approach and have the classic bio-psycho-social model. […] So I think possibly we’re at a massive advantage where the question of the permanency of lack of capacity comes in.”* Clinical psychologist

***Question 2. Presumption of capacity***

**Theme 2a. It reminds me that all decisions are different**

Most participants thought it good that the law promotes autonomy via the presumption that people have mental capacity until shown otherwise (s.1 MCA 2005). Some participants felt it was also useful as it encouraged assessing professionals to look afresh at each specific decision which an individual faced. However, there were concerns about whether the presumption worked well for decisions involving more serious consequences:

*“It’s a very broad statement. So, the nature of the specific decision that you’re looking at – trying to appreciate that presuming capacity to take 20 quid and go to the shops – is very different from assuming capacity to be able to sell the house and give all the money away to someone who’s perhaps suggesting that’s a good idea.”* Lawyer

And,

*“It’s the presumption of capacity that's decision specific. Not ‘they can sign a lasting power of attorney so I can decide what they want to eat’, because somebody might not be able to decide what they want to eat, but they can sign a lasting power of attorney.”* Social worker

**Theme 2b. But I’m only assessing them as I think they might not have capacity!**

Some professionals thought there was an inherent challenge in applying the s.1 MCA 2005 presumption of capacity. With assessment being initiating only for individuals where a concern had arisen that capacity might be lacking, professionals sometimes found it hard to put that suspicion aside. Additionally, there were occasional concerns that the presumption was exploited to justify not needing to assess capacity. This was seen as a way to reduce the workload and costs of assessment itself, but also as a way to avoid needing to manage a person who might be found to lack capacity.

Some participants had concerns about whether it was necessarily desirable for the law to promote autonomy via the presumption.

*“…it goes both ways. And I think not considering somebody might lack decision-making ability and being too presumptive, is as great a sin as invading the person’s right to make a decision and not doing a proper mental capacity assessment to justify that”* Social worker

And,

*“And the third principle causes me a little bit of concern because again, it’s not qualified. It’s just… ‘no- one should be treated as lacking mental capacity, mainly because they want to make an unwise decision’. Actually, an unwise decision may be as a result of incapacity, but equally it can be as a result of undue influences, pressures being placed upon someone. And for me, whenever someone is going to be making a decision, which on the face of it looks on unwise, it’s one of those things as a professional, you should put the brakes on. You should address: do they have the capacity? What are the other influences that are going on here? And have I done anything to mitigate that influence in my environment?”* Lawyer

**Theme 2c. I do a low intensity assessment but then get an expert in**

Some participants described a multi-stepped process to assessment, with an initial “soft” or “informal” assessment occurring, the outcome of which determined whether a full, “formal” assessment happened. Definitive assessment was sometimes seen as only possible by a professional with specific expertise.

*“And particularly, I think because often as social workers, you’re not going to get called in to do a really easy capacity assessment because someone else would have done that and it might not need something formal. It’s not going to necessarily need a proper, ‘we’re going to sit down and assess your capacity’. If it’s a smaller thing, that’s not necessarily going to impact on their life, it would just be put off until maybe they were feeling better or made into their care plan about what their wishes and how staff could help them. So when you’re doing a formal capacity, it’s because someone suspects that you may need to do something to restrict someone’s liberty or to make a choice in their best interests.”* Social worker

***Question 3. Structured testing***

**Theme 3a. They paint a useful picture of the cognitive context**

Despite some concerns about the limitations of psychological and other structured testing, many interviewees were keen to exploit whatever information might be available, to help improve their assessment. There was significant interest in cognitive profiles as providing helpful background about abilities, although there were caveats about how results should then be used.

*“There’s also the danger of being overly reliant on neuro-psychological test scores, particularly in certain areas like executive functions, which often people – when they look at the third component of the assessment of mental capacity, that ability to weigh up information as part of the decision-making process – that often correlates with performance on executive function tests. But, of course, there can be times when a person performs very well on executive function tests yet clearly, to those people that know the person, they clearly have a dysexecutive profile. And so sometimes it puts the assessor, the neuropsychologist at a disadvantage when they’re essentially trying to make the argument that a person has dysexecutive behaviour that would interfere with their ability to weigh up a decision, and yet actually the psychometric evidence is indicating that the person is fine in that way.”* Clinical psychologist

And,

*“So I find the forms a little unhelpful in that it lends itself to being a bit prescriptive in terms of having a diagnosis and then finding out the other stuff. Whereas actually you should flip it. Actually then, the psychological evidence can be useful because then you have to think a bit more creatively about why someone can't understand the relevant information. And it might be this diagnosis that you found, but it might be something else. Or it might be, they were sleepy that day or it might be they've got a UTI* [urinary tract infection] *or something. But if I find the forms, not very useful for that.”* Lawyer

Some professionals specialising in brain injury had concerns that office-based capacity assessments did not reflect real-life decision-making. Cognitive testing could be incorporated into capacity assessments to generate a more valid outcome.

*“For some, you get a gut feeling one way or the other, but you can’t see the clear evidence of the reasons why. And so in those cases, we, we would probably look at cognitive testing, particularly things like executive functioning. And all of the skills that fall within the executive function remit, to what are the indicators of whether they’re present or not? Because some people can develop such good skills that they mask the extent of their cognitive ability. They mask the impact of the learning disability. And again, you’re at risk of leaving people vulnerable to not having the support that they require or that they would want on the back of the fact that you’ve presumed capacity.”* Other health professional

Pre-assessment cognitive testing might also be used to suggest ways to support the person in the capacity assessment, in line with s.1 MCA 2005.

*“Being [at] the stage that we’re at in rehab, I use the RBANS* [( Randolph *et al.*, 1998)] *a lot and executive tests’. So somebody can do poorly on cognitive tests and still do well in the capacity assessments. And so what I’m looking for with those cognitive assessments is twofold. Partly to look at what support the person would benefit from. So if they’ve really strong visual memory, but really bad verbal memory, I might use Talking Mats* [( Murphy *et al.*, 2005)] *, not for language, but for mapping out and showing them and supporting memory. So it guides me in what help someone might have, and sometimes it’s back-up evidence to what you’re seeing.”* Psychologist

**Theme 3b. I leave that to the psychologists**

Some participants (who were not psychologists themselves) pointed out that they deferred to the expertise of psychologists. Psychologists should be the ones to administer the majority of structured testing in the most consistent and reliable manner and would therefore be the professionals best placed to comment on its relevance and use. However, there were indications that some psychologists might be cautious about the usefulness of testing:

*“I went to a meeting the other day and it was a girl who’d had a head injury some years before. And there was a lot of anxiety involved. And it was a question of her cognitive ability whether she had capacity. I’d actually said, ‘look, I want to have the psychometric tests.’ And interestingly, the psychologist said, ‘look, they’re not going to be that good because she’s not that reliable, this girl, and I don’t think the psychometric tests are going to answer it.’ So, you have the psychologist who is saying that the psychometric tests were no use. And the psychiatrist saying I want the psychometric. It was all topsy-turvy really.”* Psychiatrist

**Theme 3c. They can’t get at the actual legal criteria about decision-making**

Indeed, some voices were concerned that psychometric testing would not be able to provide definitive outcomes on capacity as described in the MCA 2005, relating cognitive profiles to performance on specific decisions.

*“I suppose they’re tests from different domains, MOCA* [Montreal Cognitive Assessment ( Nasreddine *et al.*, 2005)] *, Mini-Mental State Examination* [( Folstein *et al.*, 1975)] *– they’re medical examination skills. The assessment of mental capacity: it’s a legal requirement, so they’re just completely different domains and it’s difficult to jump from one to the other.”* Social worker

And,

*“It’s not about whether they can answer questions on, as I say, taking seven away from a hundred all the time. It doesn’t make any difference. And I see doctors rely on that a lot. Whereas capacity assessors, people who do that, we don’t rely on it. We rely on our skills and our ability to understand the situation that, that person is in.”* Other health professional

The four components of decision-making function in s.3 MCA 2005 were also seen as hard to assess in a standardised way.

*“And in terms of the person’s ability to weigh, you have to ask questions to postulate alternatives, to see if they can take the idea for an alternative, to explore whether there is any reason. I know that an unwise decision is not a reason to declare somebody incapacitous, but even an unwise decision must have some sort of rationale to it. So, one is used to exploring that that sort of thing. But as far as I’m aware, I’m not aware of any scales that help one to do that.”* Psychiatrist

There were also concerns about how useful testing could be when assessments needed to be carried out for emergency decisions or in unexpected circumstances.

*“I don’t think that would work in my specialty…. They’re already on opiates. They’re often really sleep-deprived when they’re in labour, and so their ability to pass psychometric tests would be massively impaired. Their ability to make a decision about whether they agree to a C-section or not, they’re probably capable of that, but not to do a psychometric test and it would be completely inappropriate for me to go in and start offering people psychometric tests in a labour ward consultation when someone’s in labour, frightened, vulnerable, and then you’re challenging them with a test. I don’t, yeah, that wouldn’t work.”* Doctor (non-psychiatrist)

***Question 4. Undue influence (1: presence)***

**Theme 4a. It’s our prejudices which draw the line at what become undue**

Reassuringly, professionals reported that concerns of significant malign influence or coercion were rare during their capacity assessments. Detailed discussion of these rare instances seemed to be hampered by a more over-riding concern that the concept of undue influence was too poorly defined for practitioners.

*“I don’t think it is [paid enough attention] because I’m not sure how you ever would. I’m not sure how you would ever do that because you’d have to define what undue influence is, wouldn’t you?”* Social worker

Many emphasised that a reasonable level of influence or persuasion is part of everyday life and should not be considered undue. Some expressed fears that the concept of undue influence could be misused if applied too liberally in clinical practice. For example, one professional gave an impression from their work with young people:

*“Basically undue influence is a bit of a clinician’s way of saying I don’t agree with what the parents want.”* Clinical psychologist

Some wondered whether the assessor themselves could be exerting the most dominant source of influence and thought this should be considered at times to be a form of undue influence. Drawing an analogy with assessments for compulsory psychiatric admission until the Mental Health Act 1983 (2007), one professional said:

*“So, I admit people to hospital under the Mental Health Act and when we’re talking to them, we will usually offer them informal* [voluntary] *admission. And they’ll know perfectly well, probably, that if we’re sufficiently concerned about their mental health… if they don’t accept informal admission, they’ll end up being detained. And so that’s inherently coercive, isn’t it? Even within the assessment.”* Other health professional

Many also stressed that families and friends should be presumed to be part of a healthy support network, since their views could assist assessment. Additionally, entering into a process which alienates family and friends may not be the best outcome for the person overall.

*“I get concerned about it sometimes where professionals, they start to talk about conflict of interest. And what they mean is that this person in the person’s life has a view, which differs from that of the person. And I don’t think that is a conflict of interest. That’s normal life. So I think sometimes this discussion about undue influence can get shaded into expectations on the part of professionals about almost people’s carers and friends and supporters, not being allowed to have a view or to express it to the person. I think this stuff has got to facilitate real life decision-making, not be opposed to it.”* Social worker

**Theme 4b. Capacity assessment doesn’t involve assessment of undue influence**

There were a range of views on the relationships between the common law construct of undue influence and capacity assessment. Some professionals collapsed the idea of undue influence into a broader notion of vulnerability and safeguarding concerns, as encountered in other areas of practice.

*“Okay. Golly. That’s a really hard question. I think the capacity bit is the middle bit and the voluntariness is another whole bit that’s not capacity. Because you can be competent, you can have capacity sitting in the room with me on a Tuesday. But actually, this is the victim of domestic violence, isn’t it. They can sit in the room on a Tuesday with somebody and perfectly well argue that nobody in their position should live with somebody who’s so horrible to them. Then they’ll go home. So I think that I think the two are separable. I don’t think that voluntariness is a bit of... well it’s a bit of decision-making, but it’s not about capacity.”* Clinical psychologist

Some expressed concern about the people who are deemed to have capacity but have been subject to influence or coercion, as they cannot benefit from the protections of the MCA 2005.

*“I’m sure it isn’t [appreciated] enough. I’m not sure necessarily… It’s difficult to say whether that’s part of capacity. Maybe if they lack capacity, then they’re more likely to be influenced. But sometimes I see people and they’ve certainly got capacity, but they are influenced unduly by a whole variety of pressures.”* Psychiatrist

And,

*“And do you know what I suspect is a bigger problem is the capacitous adult who is exposed to undue influence. And what do you do there? You fall back on the inherent jurisdiction. What I do is seek out advice from my solicitor, but it seems to me that there’s probably quite a lot of these people. They check all the boxes in terms of the Mental Capacity Act. They’re capacitous, particularly in terms of managing money, but they’re still being ripped off.”* Clinical psychologist

Others felt that assessors of mental capacity were discouraged from thinking about influence and coercion because these concepts are not part of the language of the capacity test in MCA 2005.

*“I don’t think it is [paid enough attention], because people [assessors] rote learned the kind of like ‘impairment or disturbance’ and then ‘understand, retain, use, weigh’, and that’s it. They know the kind of presumed capacity, people can make an unwise [decisions]. So people rote learned to lists. And I think, because undue influence and coercion aren’t on those lists, then they don’t pay attention to it, because it’s not on that kind of checklist.”* Clinical psychologist

Echoing this sentiment, some professionals gave other reflections about how undue influence might be related to mental capacity. For example, in borderline mental capacity cases, there was a question as to whether severe coercive control could itself be seen as the impairment causing functional difficulty with decisions.

*“We’re all allowed to make an unwise decision. If your unwise decision is because you’re being subject to coercion or undue influence, shouldn’t it just reflect that? And is there an argument, I suppose that the link can- maybe you might feel that there is a link- that if I am under coercive control, is there an argument that, that coercive control is somehow an impairment or disturbance in the functioning of the mind or brain? Because actually I’m not thinking straight, am I?”* Lawyer

**Theme 4c I think about it most for financial decisions**

The undue influence questions brought forth a range of case discussions. The most common concerns were around consent to make everyday financial decisions, to make a will (although this sits outside of MCA 2005), or to appoint a lasting power of attorney.

*“…and was clearly trying to get his money. But it was incredibly difficult to get to the bottom of it and to get her out of his life. Particularly at the end, she was involved in every decision. She excluded the family. And we see this sometimes. And for our patients as well. You never know when somebody comes with the best of intentions and appears to be superficially entirely trustworthy and acting in that person’s best interest because they’re loving and caring. You don’t quite know what their motives are. Then you do hear about these awful stories in the newspapers, but they exist.”* Psychiatrist

And,

*“So obviously I do financial capacity assessments because there’s been concerns about the way the family are managing it. It’s not always being done for the best outcome for the client. There’s some mutual benefits for family in the way that they manage the finances, or they’re just quite controlling. They will say ‘we need to save money for them, so they can have 20 pounds of their benefit a week’, when you know, for example, they get 70. So again, when you know that potentially there’s going to be a hostile reaction to the outcome of your assessment, you’ve got to be a little bit aware that you're following the process properly, you’re evidencing it. But you resist that pressure. Now and again, and this is unspoken, but you get the feeling the other professionals don’t want the boat to be rocked.”* Social worker

Where there was a previous history of abuse, undue influence was often at the forefront of assessors’ minds, as might be the case with some other groups seen as particularly vulnerable, including people with learning disability or brain injury.

***Question 5. Undue influence (2: support)***

The importance of seeing people alone seemed obvious to most. Although some worried that ejecting other people from assessments could be confrontational, most professionals recognised its importance and so were happy to do this.

*“Because of the… unfortunately, domestic violence and human trafficking being pretty common actually, most people in my specialty have an ear and an eye open for that and always well aware of who’s in the room with them. I make particular efforts to make sure I talk directly to the patient and only the patient. And if someone is overbearing or trying to have undue influence, I will speak to the patient alone, or I just point out that it’s the patient’s decision and that she needs to make the right decision for her in that moment.”* Doctor (non-psychiatrist)

However, some professionals argued that physically removing the person from a coercive environment is not always sufficient to mitigate the suspected influence during an assessment. They mentioned that decision making support could include longer-term relationship building, psychological interventions and access to advocacy.

*“She technically has capacity, but she doesn’t even want to stop seeing him. She’d already been removed. So, what then are you going to do? Stop someone seeing someone forever? And yes, she probably was under that much coercion, but it wasn’t actual coercion because he wasn’t anywhere near her and making no contact with her. How could you ever remove that, without basically doing serious counselling with that person? And that’s kind of where another issue lies, isn’t it, because we would have to do loads and loads of work with her. And there’s no services available to do that.”* Social worker

**Theme 5b. I gather as much information as possible**

Professionals were generally proceeding quite carefully when they intervened in suspected coercion or undue influence, as excluding friends and family members could easily be counter-productive. Talking to further family members and friends might provide useful information about the relationships in question. For some, a suspicion of undue influence initially prompted the assessor to re-consider whether they had done a thorough capacity assessment, to minimise their doubts about the outcome of that assessment.

*“So I was asked to assess capacity. So I started off with the two of them together, because I was very interested in the interaction between them. And she was very controlling, and she said ‘Nah, not now, darling. You decide whether you want me to stay in here or not [name]’. And I said ‘No, I decide, and I want you to leave now’ and off she went. And you know what, interestingly, at the end of it, I thought actually, he does have capacity to make that choice to give a chunk of money. I may not like it, but it's his choice. I think it’s a very unwise decision, but it’s a capacitous decision.”* Clinical psychologist

Other professionals thought that the absence of clear evidence about coercion meant it could be best to presume there is no undue influence.

*“And obviously, unfortunately, sometimes we don’t have that evidence. If we don’t have the evidence, we can’t assume that there’s a coercing relationship. The assumption should be that the relationship... it’s like the presumption of capacity. Presumption of a non-pathological relationship. I think, unfortunately.”* Doctor (non-psychiatrist)

**Theme 5c. I haven’t thought about it much…where’s the guidance on it?**

A number of professionals seemed not to have considered the issues in detail or felt it was too rare to need consideration.

*“I can’t think that I’ve really considered that. When I’ve been making a deputyship… I can’t think it... the decisions that we’re assessing capacity for, often it’s residents in care, finances, or admission. It’s not really in those decisions. I suppose it would only be really in safeguarding.”* Other health professional

Several lawyers had received specific training on undue influence and tried to keep up to date with CoP case law, as did some non-legal professionals. Other non-legal professionals said they had not had specific training on undue influence, with some relying instead on their own intuitions about how to proceed best.

*“I think I’ve adopted my own coping strategies to avoid it in terms of the way I would do a consultation. But I’m not sure I’ve seen any guidance or any training.”* Doctor (non-psychiatrist)

***Question 6. Responsibility and sharing assessment***

**Theme 6a. Isn’t pooling knowledge, views and responsibility what we already do?**

Some participants thought it a given that capacity assessment is best done by a multi-disciplinary teams, since working this way brings benefits to many other situations. The arbitration of disputes would not be an obstacle to the goal of integrating multiple views from people with differing skillsets.

*“Isn’t that best practice already? I thought any assessments, really any intervention that we deliver where we work in multidisciplinary teams - so any complex sort of intervention - should involve the relevant people in the multidisciplinary team, whether it’s the capacity assessment or any other kind of assessment, really. So, I can’t think why on earth, you wouldn’t work with the most appropriate people in your team to do the assessment?! That sounds a bit silly.”* Clinical psychologist

**Theme 6b. You shouldn’t pretend you can diffuse responsibility for decision-making**

Others pointed out that the law put the outcome of assessment on the shoulders of an individual, who is ultimately responsible.

*“You do need to be careful not to pick and choose between the assessments, that sort of thing. But I think my view is you don’t consult about mental capacity assessment: you just do it. You need to research, you need to take on board what the factors are in the assessments, and this is sometimes where you get a different view on factors in the assessment.”* Social worker

Professionals who regularly provided consultation on mental capacity had differing views on how easily the (referring) decision-maker could integrate the outsourced, external assessment into a final capacity outcome.

**Theme 6c. Nice idea, but it wouldn’t work when time is of the essence**

Social workers and clinicians alike commented that joint assessments might involve more time and resources than they could practically offer. In community settings, co-ordinating such an assessment was seen as a time-consuming and lengthy process of days or weeks. In emergency medical settings, a requirement for assembly of a multi-professional team was in fact seen as unrealistic.

*“I think it would be difficult in practice, because to get two people to agree, or maybe not agree on a conclusion about someone’s capacity. A lot of what we do in obstetric - I’m just thinking of that on the labour ward, where things can happen very quickly - a lot of the care is very acute. If you had involvement from different teams that might be logistically difficult.”* Doctor (non-psychiatrist)

***Question 7. Remote assessment***

**Theme 7a. It has its limits, but don’t assume all vulnerable people dislike technology**

Although carrying out remote assessments dealing with issues of mental health and cognition will bring multiple inherent challenges, some participants could not think of special considerations needed for capacity assessment in particular.

*“I can’t think of anything. I think all of the things about doing any kind of remote assessments with people with learning disabilities, whether it’s a capacity assessment or a kind of needs assessment or anything. I can’t think of’ anything very specific about capacity assessments that would be very different.”* Clinical psychologist

Although professionals were sometimes concerned about whether remote assessment would create problems for vulnerable people, equally often, remote communication formats seemed to facilitate a more relaxed assessment:

*“Some people say they prefer the kind of talking to a professional within the comfort of their own home, as opposed to seeing them in an office or something, without having the person in their home, which can feel like a bit of an intrusion. So some people might prefer it.”* Clinical psychologist

And,

*“a lot of them happened in a ward round or, in a clinic room. And actually that is just as removed from that person’s place as a virtual meeting, in a way. So whilst I can see there are probably some unique difficulties with capacity, I actually think that maybe the situation might be slightly easier.”* Psychiatrist

Indeed, remote assessment was seen as very useful for some people with significant impairment who had acquired technological fluency in response to the COVID-19 pandemic.

*“My other observation is that some people have quite a stereotype that older people will be less comfortable with technology, which is true because I’m old and I’m not comfortable with technology. But I think the fact that everybody in the country has been on a sort of a very steep learning curve. There are people in care homes now who are used to interacting with the family by remote means. So I’m not advocating that’s what we should do forever and ever. But I think we need to be careful that we’re not sort of saying Mrs. Jones is in her eighties, she won’t know what Zoom is. There still are some talented silver surfers out there.”* Social worker

**Theme 7b. I can meet them more than once now**

Travelling for assessments was seen as time-consuming, so the remote modality meant assessment could be carried out sooner, especially is juggling a long waiting list of assessments. It made it easier to involve multiple professionals or to meet the person more than once in the course of a single assessment.

*“It is also a much more effective way of managing a diary. Before COVID, my diary was booked up literally months in advance. Now if someone says, look, I need you to do an hour’s video interview over the next week. ‘Yeah, all right? I can find a time to do that’. And that was impossible pre- COVID.”* Clinical psychologist

This also makes it more likely that an assessor will be able to re-schedule an assessment at a time better for the person being assessment, thereby offering them support to demonstrate retention of capacity (s.1 MCA 2005). But some participants still wondered that face-to-face work might be superior for more complex assessments.

*“So you could probably do part of the assessment, couldn’t you, virtually, like gathering some of the information or talking through some of the scenarios. And then, if it wasn’t abundantly clear, if it wasn’t a really clear cut, non-contentious decision, then you’d probably want to do a visit as well at some point. But you could do a lot of it virtually maybe.”* Social worker

**Theme 7c. It’s tricky to pick up on environmental cues or coercion**

There were concerns that face-to-face observation of the person navigating their environment provided useful information about mental capacity. Additionally, a specific, legally relevant, worry was that the remote modality made it impossible to know definitively whether a person was alone during the encounter.

*“I think COVID and remote assessments have presented real issues in terms of undue influence. Because you and I are on the screen now, but I don’t know who’s in the room with you behind you. Somebody like whispering in your ear. So that was something that I’ve been alive to, during these strange times where we’ve done a lot of Zoom or Teams calls.”* Lawyer

## Discussion

This interview study of practices around some of the more uncertain areas of mental capacity assessment captures professionals’ views across health, social care and legal settings outside the courtroom. Our sample size was large, compared to other studies, and involved a spread of professions, generating 21 themes which retain heterogeneity of perspectives. Participants who were practice- or research-based experts in mental capacity offered crystallised opinions formed from years of consideration. These sat alongside views from non-specialists working in stretched clinical and social care services, whose views may correspond with how the bulk of mental capacity assessments are carried out. We therefore gained pragmatic insights into obstacles to assessment which must be accommodated and anticipated by policymakers.

### 1. Profession-specific issues

Members of different professions bring different training and collections of skills, performing mental capacity assessments in diverse settings and for a wide range of decisions. Any recommendations for assessment practices must work across these situations and for the approaches of healthcare, social care and legal professionals alike. We were reassured that many of our participants emphasised the need to construct a holistic picture of a person’s life.

Some non-medical professionals felt they needed a diagnosis made by a doctor before they could undertake a capacity assessment. Some participants thought that the courts already take this view, which has also been described in an analyses of CoP judgments ( Case, 2016; Series & Herring, 2017). This raises the question of who can ‘diagnose’ for purposes of s.2 MCA 2005 and link this to the functional abilities, i.e., who has sufficient understanding of that impairment of brain or mind to be able to argue how this might or might not be causing a s.3 functional inability (the so-called ‘causative nexus’)? This issue could create practical difficulties for services with a range of professionals. It would therefore be useful to have guidance produced on what the ‘causative nexus’ means for purposes of capacity assessment in order to help professionals less trained in impairments of mind/brain.

### 2. Presumption of capacity

The law is clear that assessment must start from a position of autonomy and self-determination, with the presumption of mental capacity enshrined into s.1 MCA 2005. We found that this presumption was indeed often seen as helpful, including to remind assessors to approach each decision with fresh eyes. Some participants felt uneasy that the instigation of an assessment at all might be a paternalistic act running counter-current to the presumption. However, others articulated that it was right to query capacity in high-risk decisions or where there were welfare concerns, so a risk-sensitive approach need not be seen as overly paternalistic. The drafted new MCA 2005 Code of Practice – derived after a recent governmental consultation – highlights the challenges when thinking about the presumption ( United Kingdom Government, 2022). It draws a distinction between “considering” and “assessing” a person’s capacity status, giving a route to query legitimately a capacity status without violating the presumption (at section 4.6 of the drafted code). This may not allay concerns about perverse drivers around use of the presumption to avoid offering assessment at all, which may need to be addressed via a systems-wide approach. Training and resource development might thus be directed to managing uncertainty by applying a risk-sensitive model of capacity which aims to respect autonomy, by maximising, for different gravities of decision, the chances of getting the capacity assessments right ( Berens & Kim, 2022).

### 3. Structured testing

We elicited a range of views about the use of structured or psychometric testing, but found a notable absence of reference to any research measures of capacity (such as the MacCAT-T ( Grisso *et al.*, 1997)), suggesting that these instruments are not penetrating practice. The British Psychological Society provides guidance on how neuropsychological testing can inform capacity assessment ( Herbert *et al.*, 2019), but other professional groups may not be seeking to integrate testing so directly. Although many of our participants welcomed the principle that neuropsychological instruments might provide useful adjunctive information, testing was not generally seen as able to produce definitive outcomes in cases of doubt. Some concerns echo longstanding debates about the challenges of using neuropsychological tests, such as that they usually generate a result on a scale, for which a cut-off then needs to be selected to produce a binary determination ( Kapp & Mossman, 1996). Any cut-off score will have rates of false positives and negatives which need to be optimised in measurement development, but our participants wondered whether a result which generally works at group-level might stand up to legal scrutiny for individuals – the so-called group-to-individual, or “G2i”, problem ( Faigman *et al.*, 2014). Measurement properties might be increasingly important as the stakes of the decision get higher – such as in a life-or-death decisions. Our participants also saw testing as challenging to apply in a sufficiently decision-specific manner, so capacity assessment processes may therefore need to retain some degree of flexibility.

However, it seemed that if a new psychometric measure of mental capacity could be developed to surmount these problems, then even many sceptics would receive it well. In one particular area there was notable optimism for the use of structured testing – namely with people who have impairment in executive function. There are concerns that performance during some capacity assessments for people with executive deficits does not reflect performance under real life demands ( Fisher-Hicks *et al.*, 2021; George & Gilbert, 2018; Ryan-Morgan, 2021). Our participants saw neuropsychological tests as providing useful information to arbitrate on this, and there was interest in whether office-based tests of executive function might be tailored to determine real world functioning in decisions, especially for using and weighing information. It could be that qualitative observations of performance will turn out to be of key use to psychologists, outside of the standardised testing outputs generated in neuropsychological assessments ( Thompson *et al.*, 2005). Some of these important issues have been explored in a national webinar, which includes input from a carer ( https://www.scie.org.uk/mca/directory/forum/nmc-webinars/executive-dysfunction).

### 4. Responsibility and sharing assessment

There were differing views about the use of consultation and teamwork. Whilst the law places the professional who is needing the decision to be made as the adjudicator of capacity (such as when a surgeon offers a patient an operation and seeks their consent to perform it), in practice, that professional often seems to ask for help. This might be through a locally agreed routine process of a multi-disciplinary team, or by consulting an additional professional, such as a psychiatrist, psychologist or social worker, or even an external (paid-for) mental capacity assessment service. Although this process might have parallels to that of involving expert witnesses in court determinations of capacity ( McWilliams *et al.*, 2020; Series & Herring, 2017), the idea of sharing ultimate responsibility for the mental capacity outcome itself does not exist in the law. One person is the decision-maker, and even in court, the judge is the final arbiter, not any experts who provide opinions to the court. However, some of our participants conveyed the impression that assessment responsibility could in fact be shared (or even outsourced completely). An interesting notion of “informal” or “soft” capacity assessment as a prelude to a “formal” assessment, was endorsed. This is mentioned by the English Care Quality Commission in passing ( Care Quality Commission, 2011), but is not in the law. The benefits of multi-professional co-operation when working with vulnerable people might be obvious, so one way of achieving this in capacity assessment could be via a system involving initial “soft” assessments. Changing the law to mandate multi-professional co-operation could be an option, but this would only be successful if fast, emergency assessments could be accommodated too, such as when there is immediate threat to life. In the absence of a change in the law, trainers in mental capacity assessment may wish to emphasise that the responsibility still rests with the individual decision-maker. Assessors should be encouraged to seek opinions from others, especially in difficult assessments, but then need to reflect on the opinions they receive and choose whether or not they identify with them, much as a judge does when hearing a case about contested capacity in the CoP.

### 5. Undue influence

Our results also shed light on the complex intersections between capacity and coercion. When we previously surveyed professionals, they had reported some concern about undue influence ( Ariyo *et al.*, 2021). However, in the current interview study, professionals thought that undue influence was in fact rare, even in the eyes of some experienced assessors of capacity. Some professionals were less inclined to frame relationships through a legal lens of ‘due’ or ‘undue’, preferring terms such as ‘coercive control’ ( Bettinson & Bishop, 2015) and treating influence as a broader issue which might be handled with support of a local safeguarding team. Nonetheless, professionals acknowledged that coercion or malign influence can have a detrimental impact on a person’s decision making. As such, the main concerns were not that these issues were especially common, but, rather, were about whether services could recognise situations and respond without being under or over-protective, or even coercive, themselves. The draft MCA 2005 Code of Practice uses an example case scenario to illustrate how tricky it can be, when family/friends/other people are often well-intentioned (sections 3.15-3.19) ( United Kingdom Government, 2022)

Echoing our initial survey study ( Ariyo *et al.*, 2021), we found that undue influence was most often a concern in financial decisions, as well as for contact decisions after domestic abuse, and with older adults, people with learning disability and in brain injury. Circumstances of the COVID-19 pandemic have exacerbated some of the concerns: for example, with the increase in domestic violence ( Lindsey, 2020; Piquero *et al.*, 2021) and questions around the validity of advance statements to refuse resuscitation in care homes ( Glasper, 2021; Griffith, 2020). Across the interviews, it was clear that the MCA 2005 was just one of several frameworks used in practice to safeguard adults when services suspect undue influence. Clearly, more specific training and guidance would help professionals to separate out their thinking on mental capacity and undue influence during complex assessments ( Ariyo *et al.*, 2023). This would ensure more consistent practice nationally, particularly where this has changed as a result of the pandemic.

### 6. Remote assessment

In response to the Covid-19 pandemic, the NHS in 2020 issued guidance supporting the use of remote mental health assessment ( NHS England and NHS Improvement, 2020). The courts subsequently ruled that assessments under the Mental Health Act 1983 (2007) should not be virtual ( *Devon Partnership NHS Trust and Secretary of State for Health and Social Care v. NHS Commissioning Board* [2021]) – and this was made clear in NHS guidance. However, the courts have ruled that assessment for Deprivation of Liberty Safeguards under the MCA 2005 can occur remotely ( *BP v Surrey County Council & Anor* [2020]), in line with the direction of travel of many professional bodies, such as clinical psychologists undertaking neuropsychological testing ( BPS, 2020). It seems that remote assessment of mental capacity has permeated different services to varying extents. Given the range of accounts of benefits and drawbacks, it may be difficult to draw up more specific guidance until head-to-head studies of remote and face-to-face assessments are carried out. Generic principles of good mental health working may need to be applied, together with a preference for in-person meetings for the most difficult assessments. Yet although there are important considerations for identifying undue influence remotely, these may not be so peculiar to working within the MCA 2005, as similar difficulties are found elsewhere in remote healthcare.

### Strengths

The design built on our earlier exploratory study ( Ariyo *et al.*, 2021), developing these preliminary findings and targeting the most relevant issues, using an analytic method designed for healthcare policy and where prior issues have already been identified for further exploration. Several of our findings are in line with the drafted new MCA 2005 Code of Practice, derived after national consultation ( United Kingdom Government, 2022). Compared to other interview or focus group studies exploring mental capacity assessment, our sample has the greatest mixture of professional groups, including some previously under-represented groups. The sample involved both capacity specialists and non-specialists. This means that, as well as including detailed opinions shaped by years of consideration and experience, our findings involved views of “jobbing” professionals and those working in generic settings, across which we hope our results will therefore generalise. We also believe this should make our findings of interest to policymakers.

### Limitations

Our sample did not involve service user, carer or lay perspectives. The input of these groups is essential for a more comprehensive analysis and comparison in future, although we are also unaware of involvement of these groups in studies run by other researchers.

Necessarily, not all professional groups were represented in our sample. Other researchers are better placed to comment on profession-specific issues, such as for speech and language therapists ( Borrett & Gould, 2021) or community nurses ( Cliff & McGraw, 2016), although we note that our own aims were not to make profession-specific conclusions or recommendations. This also limits the generalisability of our findings across all professions but also raises the possibility that key information was missed by not including, say, physiotherapists, pharmacists, paramedics or paediatricians.

The data used in this research were generated entirely from participant self-reflection, rather than via observing any actual practices. Participants may have only limited awareness of their own assessment practices or may have been biased in their descriptions, such as by ascribing to themselves the practices they aspired to achieve.

### Recommendations for future research

As a priority, researchers should involve the views of service users, carers and the general public in future research, seeking to identity the highest priority areas for service users and carers, and comparing results with studies like the present one. There could also be a focus on the six areas of uncertainty which the professional groups have identified. Future researchers should plan prospective studies observing professional practices directly – ideally in the real world as they take place – rather than relying on introspection and self-report.

Since the scope of the MCA is very wide, in effect including all citizens as potential assessors, we might wish to involve a very broad range of professional groups, such as the police or those working in education. Professionals who work with subgroups may have particular considerations, such as when working with specific medical conditions. The effect of assessee age also needs further research, since comparatively little work has explored the under 18 age range. Expanding the breadth of information gathered in this way would assist researchers and policymakers to ensure that all who use the MCA 2005 are included, helping us understand real world assessment practices and consider how these might be improved.

## Conclusions

We sought to illuminate capacity assessment practices in six areas of uncertainty across a range of professionals in health, social care and legal settings. Based on our interview data, some modest recommendations are made.

Policy will only achieve its aims if it works in spite of these uncertainties, within the practical constraints and competing demands faced by professionals in complex systems. For example, if capacity assessment frequently takes place using consultation and multi-disciplinary teams, then this should be acknowledged as occurring so that it can be handled in the best way. Similarly, development of guidance on remote assessment could mitigate against concerns about quality control of assessment and help to spot undue influence.

Mental capacity processes must be versatile, equally applicable in a variety of routine and emergency settings, across diverse decisional types, for both generalist and specialist assessors, and able to handle attempts at coercion. They must, ultimately, hold the autonomy of the service user centrally.

## Patient and public involvement

This study was completed within the Mental Health and Justice Project, and informed by the expertise of a service user advisory group at the McPin Foundation, whose insights, suggestions for improvements and support we are grateful for.

## Ethics and consent

Ethical approval was achieved from the King’s College London’s College Research Ethics Committee on 11/02/19 (MRA18/19-10500). Written informed consent was received for participation, supplied electronically and in line with our ethical approval.

## Data Availability

In line with the terms of the ethical approval for this project, as provided by the King’s College London Research Ethics Committee, we are unable to provide unrestricted access to the full dataset, due to concerns that this would make some participants identifiable. Restricted data is available on reasonable request. This is likely to be a request from researchers for the purposes of further research, and may require submission of a detailed study protocol and the signing of a data access agreement. Open Science Framework: SRQR checklist and flow chart for ‘Mental capacity assessment in the multi-professional real world: a qualitative study of six areas of uncertainty.’ https://doi.org/10.17605/OSF.IO/RFHKN ( McWilliams, 2023). Data are available under the terms of the Creative Commons Zero “No rights reserved” data waiver (CC0 1.0 Public domain dedication).

## Software availability

We used https://www.descript.com to perform our initial transcriptions, which we then listened to by ear and corrected manually. Although this is a paid-for service, a number of free transcription services are available, such as https://get.otter.ai/interview-transcription/?keywordgroup=free+transcription.
