# Supplementary material for: Mental capacity assessment in the multi-professional real world: a qualitative study of six areas of uncertainty
Source: Wellcome Open Res. 2024 Apr 24;9:221. [Version 1] doi: 10.12688/wellcomeopenres.20952.1 (PMC11190650; doi:10.12688/wellcomeopenres.20952.1)
Supplement: Peer review history

# Reviewer report: Reviewer response for version 1

**DOI:** 10.21956/wellcomeopenres.23179.r84569

**Published:** 2024-06-21

**Authors:** Kevin de Sabbata

**Affiliations:** 1 Keele University, Keele, England, UK

Dear colleagues,

 I have really enjoyed reading this interesting study. I think the research provides some really valuable insights into the practice of mental capacity assessments, which really add to existing knowledge. Below you find a few pointers which would help you to present your findings and the big messages of your research even better.

- Page 4, paragraph 2. I am not sure I would define George Smuzkler as a ‘human rights scholar’.

 

- Page 4, paragraph 4. As you cite the UN Convention on the Rights of Persons with Disabilities (CRPD), I would also cite the fact that the CRPD Committee and a series of scholars criticise the very same concept of mental capacity assessment, arguing that mental capacity assessments are not impartial and can often seem unreliable and prone to prejudice. You cite the point (made by Kong and Scholter and Gather), that supported decision making could be the means for people around the incapable person to project their own interests and agendas on the situation. But there is also the opposite criticism that also mental capacity assessments can be influenced by the assessor’s agenda, projecting a hyper-cognitivistic attitude on the situation. In this sense, scholars claim that substituted decision-making mechanisms provide an even bigger space than supported decision-making for people around the incapable person to further their own agendas (because there the law even authorises them to set aside the person’s subjective considerations if they can show somehow that this is in the person’s best interests). I would add this in your background section. On these aspects see, for example, [1]

- Pages 4 and 5: I would also cite the Mental Capacity Act (MCA) Post-Legislative Scrutiny by the House of Lords, as an extensive and authoritative survey of how the MCA is being implemented (and which stressed the MCA is not that well understood). It’s from around 10 years ago, but still relevant as a background source.

 

- Page 8, second column line 5. I believe it’s ‘initiated’ and not ‘initiating’?

 

- Page 11, second column 18 ^th^ line from the bottom: there is a typo on ‘pressure’

 

- An element that I see emerging from your data at page 6-8 is that everyone seems to think that their profession is the best at doing capacity assessments. Can this create situations of professional arrogance and be an obstacle to inter-professional collaboration and holistic assessments? I would briefly reflect on this in the discussion.

 

- I would reflect a bit more, in the discussion, on what participants say (for example at page 6 and 8) on the need for a diagnosis, and diagnostic skills and understanding of medical terminology to carry out a mental capacity assessments, in relation to the fact that the law in reality does not require a formal diagnosis for a declaration of incapacity.

 

- Reflect a bit, in the discussion, on the relationship between psychological testing and the parameters of the MCA. Could there be a danger that, depending on what psychological tests one selects, the result of the assessment changes? Could a certain test which focuses on some abilities/parameters and not others be seen to convey different values/norms, which might even contrast with those of the MCA? On the other hand, maybe the tension between psychometric testing and mental capacity criteria (which you cite at page 14), is also a sign of the inappropriateness of the binary concept of capacity at the centre of the MCA? There has been a lot of criticism and debate on the binary concept of capacity and on the fact that it does not match with reality, in which capacity is on a spectrum (see for example the work of Beverley Clough on this). I would reflect more on these aspects in the discussion.

 

- Many of your participants (see for example page 10) stressed that the family should be part of a healthy support network. Connect this to debates in the CRPD. There is a lot of debate about the viability and risks of involving people surrounding the incapable person in supported decision making under the CRPD, but from your study it seems this is already perceived as a requirement.

 

- Not completely clear how/where data were stored. Can you briefly clarify that?

Is the work clearly and accurately presented and does it cite the current literature?

Yes

If applicable, is the statistical analysis and its interpretation appropriate?

Not applicable

Are all the source data underlying the results available to ensure full reproducibility?

Partly

Is the study design appropriate and is the work technically sound?

Yes

Are the conclusions drawn adequately supported by the results?

Yes

Are sufficient details of methods and analysis provided to allow replication by others?

Yes

Reviewer Expertise:

Mental Capacity Law, dementia, human rights

I confirm that I have read this submission and believe that I have an appropriate level of expertise to confirm that it is of an acceptable scientific standard.

## References

[1] : Dementia, Treatment Decisions, and the UN Convention on the Rights of Persons With Disabilities. A New Framework for Old Problems. *Front Psychiatry* .2020;11: 10.3389/fpsyt.2020.571722 571722 10.3389/fpsyt.2020.571722 33240127 PMC7680726

# Reviewer report: Reviewer response for version 1

**DOI:** 10.21956/wellcomeopenres.23179.r84572

**Published:** 2024-06-19

**Authors:** Emanuele Valenti

**Affiliations:** 1 Warwick Medical School, Coventry, UK

The paper under review is a remarkable work aimed at understanding how professionals in real-world situations assess mental capacity. The authors conducted forty-four semi-structured interviews with professionals from different backgrounds. The study aims to follow up on a previous investigation about mental capacity assessment in a real-world setting carried out with an exploratory survey of 611 professionals who answered several questions related to the mental capacity assessment procedure. The previous investigation involved mainly social workers and was limited to a structured survey. The research team planned to explore findings through a follow-up qualitative study with semi-structured interviews that included healthcare professionals, lawyers, and previous social workers. The research question mentioned by the authors is oriented toward exploring processes and policy uncertainty related to six areas identified in prior research involving social workers. The paper has an undoubted quality and constitutes a significant outcome for understanding procedures related to assessing mental capacity and depicting participants' experiences. Findings offer a comprehensive view of how capacity is assessed in England and Wales and an invaluable insight into improving policies and guidance available to implement the Mental Capacity Act. The introduction provided a comprehensive description of the main issues related to mental capacity assessment procedures and the effective implementation of the MCA's provision. However, authors sometimes idealise MCA rather than present it for its actual effect on service delivery and professionals’ experience. For instance, on *p 4.  lines 3-4, left, *the authors stated that the Act promotes “personal autonomy against Best Interests”.

 Previous investigations indicate that mental capacity assessment and best interests determination may be confused or wrongly exchanged [1], and autonomy promotion does not always seem to be an MCA applicant's priority. Several professionals are unhappy with the MCA and consider it a very paternalistic Law. It is recommended that the reader be offered a comprehensive view of the challenges the law represents. Modifying the sentence with "wish to promote autonomy and to protect best interests" would be sensible. In the same vein, the authors referred to a "wealth of training provided" (p. *4 li*nes 15-18) without mentioning that training constitutes another central issue of MCA implementation in the services [2] *, *and this opinion is confirmed by two different empirical investigations in which the reviewer has been involved in 2019-2023 and 2024 for which publication are not still available. Literature reported that the training programs were unsatisfactory a decade after the enactment of the Law [3] *.*

 The paper aims to present findings related to the exploration of six uncertainty areas identified in a previous study. However, the lack of an apparent reference to the previous study's aim does not immediately make clear the circumstances in which these areas have been identified and why their exploration is relevant. The authors refer to the need for a more tailored sample in which other relevant decision-makers were better represented. Providing more specific examples and suggestions would help to match research objectives with a more straightforward description of the methodology, making the audience feel more engaged and involved in the reading.

 The authors justified the framework method as appropriate to answer topic guide interviews and explore questions about health and social care policy. Nevertheless, framework analysis in applied policy research can be used for different purposes, each defined according to the role of the research question. Clarifying research questions would help to understand which kind of framework analysis has been carried out and why, among the available approaches suggested by Ritchie & Spencer (1993), the authors made their preference.

 The qualitative analysis would have been improved by applying qualitative data analysis software to assist researchers in managing data and visualising findings. Whereas the thematic framework is consistent with topic guide questions, it seems devised mechanically, and the themes generated are underdeveloped and non-refined enough.

 Authors chartered and indexed data as described (p 5, lines 35-44, left) using a spreadsheet to summarise or reduce them. Reduction is obtained by selecting data according to each topic guide question, and each question generated three themes for a total of 21 themes. The high number of themes shows that the data has been reduced, but it could have been better mapped and interpreted. That means generating themes independently from each question and defining data conceptually. The analysis produced a suitable matrix but needed a conceptual framework that provides the critical dimensions required to associate one theme with another. What is missed in the study is a comparison between themes and a synthesis of sub-themes in units of meaning. By way of example, if we consider theme number 1, 1a-1b-1c could be the sub-themes of a more general theme defined as professional background and focused on exploring how different participants' qualifications provide a distinct concept of mental capacity assessment and enquiring if such differences are ascribable to their knowledge, to their other role in decision-making, to their professional duties, and if such differences allow to depict a different model of mental capacity for each professional group.

 Further analysis would allow a constant and systematic comparison between sub-themes and themes and a progressive refinement to clarify conceptual participants' experiences and generate meaning to inform findings. In qualitative research, summarisation is more than a reduction; it is a creative synthesis that produces new insights to drive the understanding of a social phenomenon. Authors expect "to gain pragmatic insights into an obstacle to assessment which must be accommodated and anticipated by policymakers".  In their discussion, they depicted normative content from participants' descriptions; the alignment between descriptive and normative aspects of the study is missing in the analysis. In addition to Ritchie & Spencer, suggestions for theme framework refinement could be made by Miles, Huberman, Saldana (2014) Qualitative Data Analysis, and Sage.

 A more practical recommendation for authors is a description of each quotation with a different code for each participant and their gender, in addition to the profession, to ensure internal generalisability of themes and underline gender dimension. Reducing quotation length would improve readability and help authors to summarise and refine themes. The results section currently has around 6000 words, and the standard qualitative paper has 8000 words. The number of words is a good indicator of the analytical work required to refine themes. Twenty-one themes might be the expected number of a research report, and such a number would indicate analytical accuracy. In a journal article, however, they may be an indicator of incomplete analytical work. Authors have different summarisation criteria with such a rich data set; they can refine themes for each respondent across all the themes and reduce the number of themes to   4 and 6.

Is the work clearly and accurately presented and does it cite the current literature?

Yes

If applicable, is the statistical analysis and its interpretation appropriate?

Not applicable

Are all the source data underlying the results available to ensure full reproducibility?

Yes

Is the study design appropriate and is the work technically sound?

Yes

Are the conclusions drawn adequately supported by the results?

Partly

Are sufficient details of methods and analysis provided to allow replication by others?

Partly

Reviewer Expertise:

I'm an expert in medical ethics with more than 15 years of experience in empirical research and qualitative methodology. I have been involved in a Wellcome Trust funded 5 years research project about MCA and best-interests decision-making (BABEL), and I'm currently the study coordinator of another NIHR 3 years funded project of about MCA focused on the assessment of mental capacity (HELPS).

I confirm that I have read this submission and believe that I have an appropriate level of expertise to confirm that it is of an acceptable scientific standard, however I have significant reservations, as outlined above.

## References

[1] : Best interests decisions: professional practices in health and social care. *Health Soc Care Community* .2014;22(1) : 10.1111/hsc.12066 78-86 10.1111/hsc.12066 23981053

[2] : Going home? An ethnographic study of assessment of capacity and best interests in people with dementia being discharged from hospital. *BMC Geriatr* .2014;14: 10.1186/1471-2318-14-56 56 10.1186/1471-2318-14-56 24758694 PMC4005620

[3] : Is there a broader role for independent mental capacity advocates in critical care? An exploratory study. *Nurs Crit Care* .2018;23(2) : 10.1111/nicc.12290 82-87 10.1111/nicc.12290 28247553

# Reviewer report: Reviewer response for version 1

**DOI:** 10.21956/wellcomeopenres.23179.r84577

**Published:** 2024-06-08

**Authors:** Hugh Series

**Affiliations:** 1 Oxford Neuroscience and Faculty of Law, University of Oxford, Oxford, England, UK

**As the authors rightly point out the Mental Capacity Act 2005 (MCA) reaches into healthcare, social care and legal settings. The seemingly clear wording of the Act does not always translate into simple and clear assessments of capacity. This study builds on a prior exploratory study of 611 professionals, by interviewing  44 participants (a mix of social workers, clinical psychologists, psychiatrists, other doctors, and other health professionals) in more detail and using framework analysis to examine uncertainties in six main areas identified from the previous study and elsewhere. The six areas were examined by means of seven questions (two questions on undue influence). For each question, three main themes were identified.**

**This approach gives the study not only a wide sense of how different professionals approach capacity, but also allows more detailed responses to be drawn out, often using verbatim quotes. Framework analysis is an appropriate tool to use to allow a systematic approach to an area which is inherently both very open and somewhat subjective. It is not an area which is readily studied by a quantitative analysis methodology.**

**The question of how professionals view and use MCA to make capacity assessments is both important and relatively little studied, so the present paper is very welcome.**

**Slightly disconcertingly, in the Introduction, the summary of MCA sections 2 and 3 which define incapacity is not given exactly as stated the Act, which does not itself refer to ‘decisional functioning’.**

**Although this is a study about the assessment of capacity, the authors also discuss undue influence which, at least from a legal perspective, is a separate issue. The responses highlight the close connection between the two concepts in practice.**

**A great many important observations are reported here. It is a rich source of ideas and views about aspects of assessing capacity. Organising the responses by question and then themes is a helpful way to present it. In this review it is only possible to refer to a few of the wide variety of responses.**

**While some professionals felt at a disadvantage if they were not familiar with diagnostic considerations (lawyer, social worker) others felt that their own professional background gave them an advantage for one reason or another (social worker, clinical psychologist, GP).**

**‘Undue influence’ is not a term which appears in MCA (‘undue pressure’ is referred to in connection with making a Lasting Power of Attorney, but not otherwise). The legal meaning of the term comes from case law: undue influence arises essentially when the will is overridden without convincing the judgement. An attempt to persuade the person is not the same thing as undue influence. Some respondents provided very thoughtful comments on the relationship with capacity, such as a lawyer who asked whether coercive control could be seen as an impairment or disturbance in the functioning of the mind or brain, and a social worker who argued that coercion could be exercised at a distance on a person who did not want to stop seeing the person, even after the influencer had been removed, so that it could be extremely difficult to remove the influence.**

**There was a divergence of view amongst respondents about the value and limitation of remote assessments of capacity. There can be advantages: saving of time, some clients might prefer it, the possibility of making repeat assessments. But there may be disadvantages: missing cues, lack of technological fluency, knowing whether the person is alone in the room.**

**There was also a divergence of views about the value (or lack if it) of psychometric testing. A common theme was that psychometric tests do not test capacity directly; a person who scores well on such a test may yet lack capacity, and a reliance on cut-offs will generate false positives and false negatives. However, many participants felt that tests might provide useful, if not determinative, information.**

**The nature of the study area precludes any possibility that this would offer a comprehensive view of practice, but that was not the aim, and does not detract from its relevance. The authors have sought to interview a range of appropriate professionals. As they acknowledge, not all professional groups were represented, and service users and carers were not included. Nor can it be assumed that the responses given in this study were in any way representative of each professional group. One practitioner is likely to assess capacity very differently from another, even of the same profession.**

**Although there is no ‘right way’ to conduct a capacity assessment against which practice might be compared, chapter 4 of the MCA Code of Practice discusses how to assess capacity. No attempt was made in this study to evaluate how far participants followed that guidance.**

**The study is of great relevance in designing training in capacity as it identifies many areas of uncertainty and variations in practice. It is also of relevance to policy makers. The revised Code of Practice for the MCA has been published, but is currently in draft, and one might hope that the final version will take into account the insights that this paper has generated.**

Is the work clearly and accurately presented and does it cite the current literature?

Yes

If applicable, is the statistical analysis and its interpretation appropriate?

Not applicable

Are all the source data underlying the results available to ensure full reproducibility?

No

Is the study design appropriate and is the work technically sound?

Yes

Are the conclusions drawn adequately supported by the results?

Yes

Are sufficient details of methods and analysis provided to allow replication by others?

Partly

Reviewer Expertise:

NA

I confirm that I have read this submission and believe that I have an appropriate level of expertise to confirm that it is of an acceptable scientific standard.
